# A general strategy to inhibiting viral −1 frameshifting based on upstream attenuation duplex formation

**DOI:** 10.1093/nar/gkv1307

**Published:** 2015-11-26

**Authors:** Hao-Teng Hu, Che-Pei Cho, Ya-Hui Lin, Kung-Yao Chang

**Affiliations:** Institute of Biochemistry, National Chung-Hsing University, 250 Kuo-Kung Road, Taichung, 402 Taiwan

## Abstract

Viral −1 programmed ribosomal frameshifting (PRF) as a potential antiviral target has attracted interest because many human viral pathogens, including human immunodeficiency virus (HIV) and coronaviruses, rely on −1 PRF for optimal propagation. Efficient eukaryotic −1 PRF requires an optimally placed stimulator structure downstream of the frameshifting site and different strategies targeting viral −1 PRF stimulators have been developed. However, accessing particular −1 PRF stimulator information represents a bottle-neck in combating the emerging epidemic viral pathogens such as Middle East respiratory syndrome coronavirus (MERS-CoV). Recently, an RNA hairpin upstream of frameshifting site was shown to act as a cis-element to attenuate −1 PRF with mechanism unknown. Here, we show that an upstream duplex formed in-trans, by annealing an antisense to its complementary mRNA sequence upstream of frameshifting site, can replace an upstream hairpin to attenuate −1 PRF efficiently. This finding indicates that the formation of a proximal upstream duplex is the main determining factor responsible for −1 PRF attenuation and provides mechanistic insight. Additionally, the antisense-mediated upstream duplex approach downregulates −1 PRF stimulated by distinct −1 PRF stimulators, including those of MERS-CoV, suggesting its general application potential as a robust means to evaluating viral −1 PRF inhibition as soon as the sequence information of an emerging human coronavirus is available.

## INTRODUCTION

Reading-frame maintenance is crucial for translational fidelity because it ensures that codons are in the correct reading-frame of an mRNA on delivery into the A site of an elongating ribosome. However, functional translational frameshifting is programmed site-specifically into particular mRNA of a variety of mobile elements as well as viruses and a few cellular genes (1–7). Specifically programmed sequences and structures in mRNA can cause a fraction of elongating ribosomes to shift 1 nt in the 5′-direction of mRNA, leading to a −1 programmed reading-frame shift (PRF), whereas a +1 frameshifting occurs when the ribosome slips toward the 3′-direction by 1 nt (8). In addition to the in-frame translation products, frameshifting events thus allow the synthesis of an extra protein with its N-terminal and C-terminal regions (separated by the shifting site) encoded by the 0-frame and the shifted frames, respectively. Many viruses require −1 frameshifting in their decoding of crucial viral genes and rely on −1 PRF efficiency to control the ratio between viral proteins for optimal viral propagation.

Efficient eukaryotic −1 PRF requires two cis-acting elements in mRNA, a slippery sequence (where frameshifting occurs) and an optimally placed downstream stimulator structure. An X_XXY_YYZ sequence in the slippery site facilitates −1 frameshifting by paving codon-anticodon disruption in the P and A sites of the 0-frame (XXY and YYZ codons) and codon-anticodon repairing in the −1 frame (XXX and YYY codons). This transition is further enhanced by resistance from the downstream stimulator (usually a pseudoknot or a hairpin) to the duplex unwinding activity of ribosome, leading to interference in the translocation step of an elongation cycle (9–13). Additionally, the spacing nucleotide number between the slippery site and downstream stimulator affects −1 PRF efficiency because it helps positioning the slippery site in the A and P sites of an elongating ribosome while the downstream stimulator approaches the mRNA entry channel of the ribosome (14). It has been proposed that tension is created between the unwinding stimulator and the codon-anticodon interaction network anchored around the ribosomal P and A sites, and the shift to −1 frame relieves the tension and overcomes the ribosomal pause imposed by the stimulator (14–16). Interestingly, base-pairing interaction between an internal Shine-Dalgarno (SD)-like sequence upstream of the frameshifting site and anti-SD sequence in 16S ribosomal RNA also acts as a frameshifting regulator in 70S ribosome (17,18). This could be due to the tension or a translation pause mediated by the upstream SD·anti-SD mediated duplex (19).

Mutagenesis in viral −1 PRF signals to change −1 PRF efficiency has been shown to impair the replication of several viruses, including HIV and severe acute respiratory syndrome coronavirus (SARS-CoV), suggesting that viral −1 PRF regulation is a potential antiviral means (20–22). Given the crucial role of −1 PRF for efficient viral replication, different strategies have been developed to target viral −1 PRF stimulators to explore potential antiviral applications. Small ligands capable of interfering with viral −1 PRF activity by binding with the downstream −1 PRF stimulators of HIV and SARS-CoV have been identified either by screening or structure-based design (23–25). Alternatively, antisense peptide nucleic acid (PNA) targeting the viral −1 PRF stimulator pseudoknot has been shown to impair the replication of an SARS-CoV replicon (26). For both approaches, the functional characterization of a viral −1 PRF stimulator is required and this may represent a bottle-neck in combating emerging epidemic viral pathogens such as the MERS-CoV (27).

Recently, an RNA hairpin upstream of the −1 frameshifting site of the SARS-CoV has been shown to attenuate −1 PRF depending on hairpin stability and an optimal spacer length between the slippery site and hairpin (28,29). This unique upstream hairpin represents the first cis-element capable of downregulating eukaryotic −1 PRF activity and understanding its functional mechanism should provide insight into the mechanism of −1 PRF regulation with antiviral application potential. As the upstream attenuation hairpin is unwound by the ribosome before the ribosome encounters the downstream stimulator, it has been proposed that the refolding dynamics of the hairpin is responsible for its −1 PRF attenuating activity (29). Here, we found that an RNA–DNA duplex formed by annealing antisense DNA to its complementary mRNA sequence upstream of a −1 PRF slippery site could attenuate −1 PRF to a similar extent as that of an upstream hairpin attenuator. That the cis-formed upstream hairpin can be replaced by a trans-formed duplex suggests upstream duplex formation is the determining element in −1 PRF attenuation. This finding is reminiscent of frameshifting regulation by SD·anti-SD mediated short upstream duplex in 70S ribosome (17,18), providing insight on the functional mechanisms of upstream −1 PRF attenuation in 80S ribosome. Furthermore, we apply this upstream duplex attenuator to counteract several viral −1 PRF signals to demonstrate its general application potential as an alternative −1 PRF inhibition approach. Thus, inhibiting −1 PRF by antisense-mediated upstream duplex provides a potentially quick antiviral solution to the emerging highly pathogenic coronaviruses and an opportunity to sequence-specifically regulate −1 PRF related cellular events.

## MATERIALS AND METHODS

### Plasmids and construction of reporters

We used two different −1 PRF reporters to analyze frameshifting efficiency in this study. The p2luc recoding reporter (30) was a gift from Professor John Atkins at the University of Utah, and was used in radioactivity based *in vitro* translation as well as dual-luciferase based measurement in both *in vitro* translation and 293T cells for frameshifting efficiency calculation. Additionally, a variant of p2luc was engineered to facilitate radioactivity based −1 PRF activity analysis *in vitro*. The variant contains a premature −1 frame stop codon 33 nt downstream of the BamHI site of p2luc, and will be translated into a shortened −1 frame product in reticulocyte lysate (31).

### Recombinant DNAs and mutagenesis

The −1 PRF elements used in this study, containing different viral downstream −1 PRF stimulators and upstream sequences flanking the slippery sites, were constructed by assembling different pieces of chemically synthesized DNA oligonucleotides with partially overlapping sequences via the polymerase chain reaction (PCR)-based ligation approach (32). Forward and reverse DNA primers, respectively carrying SalI and BamHI restriction sites and appropriately designed annealing sequences, were used for PCR amplification of the cDNAs encoding viral −1 PRF elements of interest. The amplified inserts encoding distinct viral −1 PRF signals were then cloned into the SalI/BamHI sites of appropriate −1 PRF reporters. Cloning was performed using standard procedures and the resultant recombinant −1 PRF reporters were trans-formed into the DH5α strain of *Escherichia coli* cells for maintenance and selection by ampicillin. Mutagenesis was introduced into the desired position using the quick-change mutagenesis kit from Stratagene according to the manufacturer's instructions. Identities of all cloned and mutated −1 PRF elements were confirmed by DNA sequencing analysis.

### Oligonucleotides synthesis and purification

Synthetic RNAs used in this study were transcribed by T7 RNA polymerase with designed DNA templates using *in vitro* transcription method (33). The transcribed RNAs were purified by 20% denaturing polyacrylamide gel electrophoresis in the presence of 8 M urea and the gels of bands containing desired RNA were cut out and electro-eluted using a BIOTRAP device (Schleicher & Schuell). The eluted RNAs were ethanol precipitated and recovered by centrifugation. Antisense DNA oligonucleotides were chemically synthesized and purchased from Mission Biotech, Taiwan, whereas the 2′ OMe-modified RNA oligonucleotides were purchased from TriLink BioTechnologies, Inc., USA. The concentration of all oligonucleotides was determined by UV absorbance.

### Human cell culture and cell lysate preparation

The 293T cells were cultured in Dulbecco's Modified Eagle Medium (Gibco) supplemented with 10% fetal bovine serum (FBS) (Gibco) on 10-cm dishes to 90% confluency. Cells were then transferred to 15-cm dishes, incubated to 90% confluency and then washed with ice-cold phosphate-buffered saline (PBS) and detached by trypsinization using trypsin-EDTA (0.05% Trypsin with 2 mM EDTA, Gibco). After stopping trypsinization with medium containing 10% FBS (Corning), the detached cells were centrifuged at 1000 *g* at 4°C to pellet the cells. The cell pellet was washed twice with PBS and re-suspended with hypotonic buffer (20 mM HEPES (pH7.5), 100 mM potassium acetate, 1 mM Magnesium acetate, 2 mM dithiothreiol (DTT) and proteinase inhibitor cocktail (Roche)). Re-suspended cell pellets were incubated on ice for 45 min and homogenized through a 1 ml syringe using 26 G, 3/4-inch needle (34). After centrifugation at 14 000 *g* for 1 min at 4°C, the supernatant containing cell lysate was collected with the contents of protein concentration measured by Bradford assay (Biorad), and stored at −80°C.

### Human cell-based frameshifting assay

Human embryonic kidney HEK-293T cells were cultured as described above. One day before the transfection, 0.5–1 × 10^5^ HEK-293T cells per well were plate in a 24-well culture plate with 1000 μl growth medium. Transfection was carried out, by adding a mixture of 0.5 μg plasmid DNA and jetPEI™ transfection reagent (Polyplus) into each well, according to the manufacturer's instructions. Luciferase activity measurements for transfected 293T cell lysates were performed as described below.

### *In vitro* radioactivity- and dual-luciferase-based −1 PRF assays and frameshifting efficiency calculation

Capped viral −1 PRF reporter mRNAs were prepared using a mMESSAGE mMACHINE high-yield capped RNA transcription kit (Ambion) by following manufacturer's instructions. Reticulocyte lysate (Ambion) as well as human 293T cell lysate was used to generate shifted and non-shifted protein products for frameshifting analysis. For radioactivity-based assay in reticulocyte lysate, a total of 5 μl reaction containing 100 ng of capped reporter mRNA, 2.5 μl of translation lysate and 0.2 μl of 10 μCi/μl ^35^S-labeled methionine (NEN) was incubated at 30°C for 1.5–2 h The *in vitro* translation samples were resolved by 12% sodium dodecylsulphate-polyacrylamide gel electrophoresis and exposed to a phosphorimager screen for quantification after drying. Frameshifting efficiencies were calculated, by dividing the counts of the shifted product by the sum of the counts for both shifted and non-shifted products, with calibration of the methionine content in each protein product. We presented all of our radioactivity-based *in vitro* −1 PRF results in term of relative −1 PRF activity and the ribosome drop-off effect (30) was removed by this procedure. *In vitro* dual-luciferase based −1 PRF assay was performed in reticulocyte lysate or human 293T cell lysate as described in each experiment. For a 10 μl *in* *vitro* translation reaction using 293T cell lysate, the reaction contained ∼5 μg/μl cell lysate and 500 ng of RNA templates in a translation buffer of 20 mM HEPES, pH7.6, 80 mM potassium acetate, 1 mM Magnesium acetate, 1 mM ATP, 0.12 mM GTP, 20 mM creatine-phosphate, 0.1 mg/ml creatine phosphokinase, 2 mM DTT, 0.15 mM spermidine and 400 U/ml RNasin (Promega) (35). The reactions were incubated at 30°C for 2 h. Luciferase activity measurements were performed using the Dual Luciferase™ reporter assay (Promega) according to the manufacturer's instructions on a CHAMELEON™ multi-label plate reader (HIDEX). Frameshifting efficiency was then calculated according to previously described procedures with read-through controls (30).

### Analysis of RNA–DNA complex by electrophoretic mobility-shift assay (EMSA)

The purified RNA transcripts were treated with rAPid alkaline phosphatase (Roche) in the presence of RNase inhibitor (Promega) at 37°C for 1 h to remove the unlabeled 5′-phosphate. After the inactivation of phosphatase by incubation at 75°C for 2 min, ^32^P -γATP (Amersham) and T4 polynucleotide kinase (Roche) were added and the reaction continued for 40 min at 37°C. The ^32^P -labeled RNAs were purified by 20% denaturing polyacrylamide gel and recovered by crush and soak procedures. After ethanol precipitation, the labeled RNAs were recovered by centrifugation. To analyze RNA–DNA interactions, labeled RNA probes (10 000 CPM per reaction) were incubated with various amounts of antisense DNA in a final volume of 10 μl of 1× TBE buffer containing 100 mM NaCl and 0.1 mM EDTA. The RNA–DNA complexes were heated at 80°C and annealed for 30 min at 30°C. The reactions were then mixed with 2 μl of 40% sucrose as the loading buffer and loaded into a 20% non-denaturing polyacrylamide gel (19:1 acryl:bisacryl ratio) in 0.5× TBE (Tris-boric acid-EDTA) run at a constant voltage of 150V at 4°C for EMSA analysis. The results were visualized by autoradiography using a Typhoon FLA7000 phosphorimager (GE).

### Statistical analysis of experimental data

Unless otherwise indicated, experiments were performed in triplicate and the relative frameshifting activity was reported as one standard deviation from the mean. Analysis of variance (ANOVA) in each set of data (without or with different amounts of antisenses) was performed. Datasets with an *F*-value bigger than the critical values from a lookup table for α = 0.05 and *P*-value smaller than α were then further analyzed by pairwise comparisons to compute the smallest significant difference (LSD) with a *t*-test. Additionally, extra experimental data sets were obtained for MERS −1 PRF assay in human 293T cell lysate with or without 10 μM of 2′ OMe-modified RNA antisense and compared with those of a read-through control (Supplementary Figure S6) to perform a statistical analysis suggested for dual-luciferase based assay (Figure 6B) (36).

## RESULTS

### Regulation of −1 PRF by controlling upstream attenuator hairpin formation

**Figure 1.**
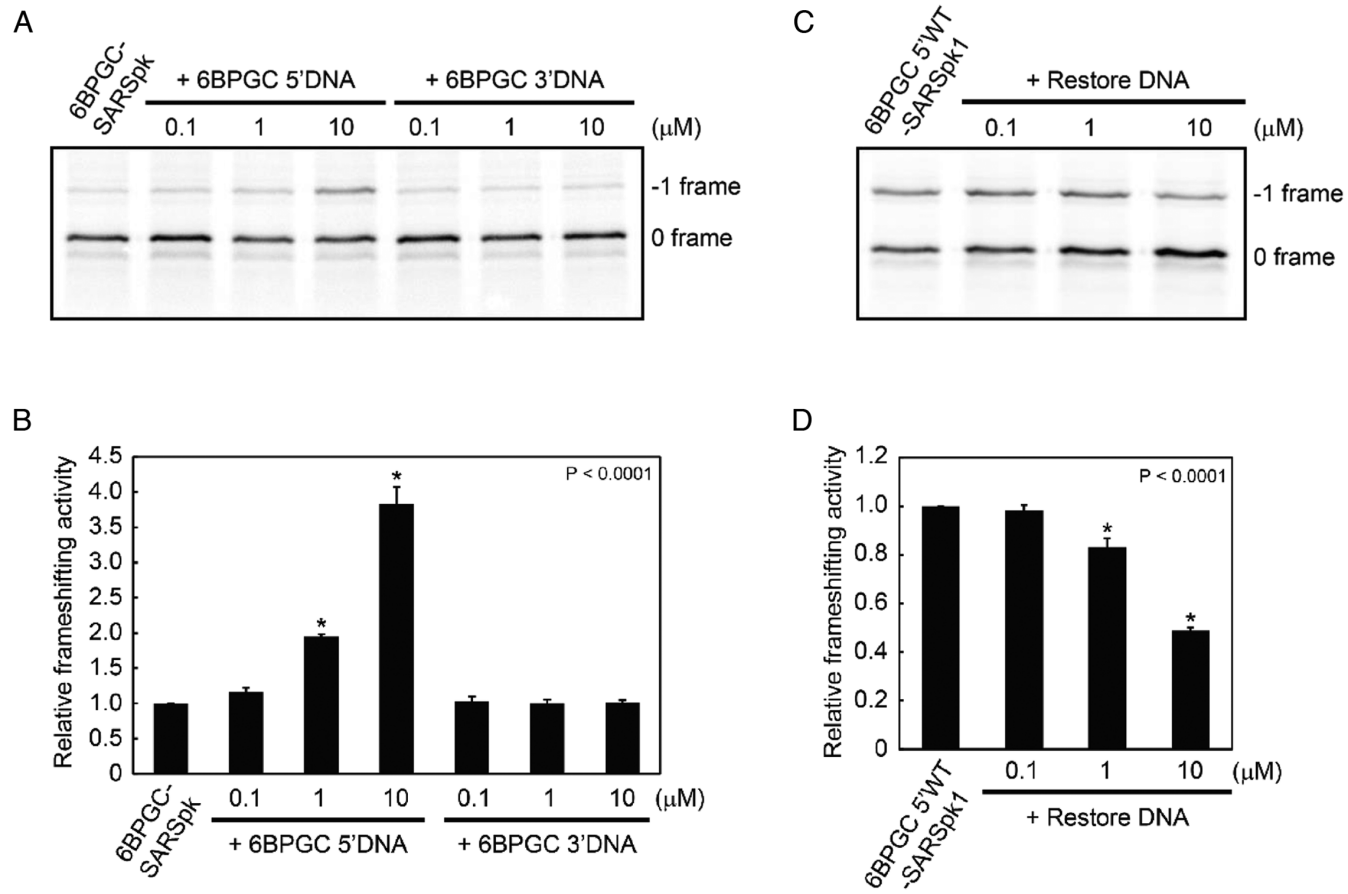
*In vitro* tuning of −1 PRF activity by antisense DNA designed to target 5′- or 3′- side of an upstream attenuation hairpin stem. (**A**) Sodium dodecylsulphate-polyacrylamide gel electrophoresis (SDS-PAGE) analysis of ^35^S methionine-labeled translation products in reticulocyte lysate using a shortened p2luc −1 PRF reporter containing an upstream 6BPGC and a downstream SARS-CoV pseudoknot stimulator in the presence of different amounts of antisense DNA oligonucleotides (as illustrated in Supplementary Figure S1A). The 0 and −1 frame products are labeled as indicated. (**B**) Relative frameshifting activity of (A) with the frameshifting efficiency of reporter without antisense DNA addition being treated as 1 for comparison. Value for each bar is the mean of three independent experiments with standard error of the mean. *P-*values were determined by a student's *t-*test with *P-*value < 0.0001 designated by an ‘*’ and referring to the comparison with the construct without the addition of an antisense. (**C**) SDS-PAGE analysis of ^35^S methionine-labeled translation products in reticulocyte lysate using a shortened p2luc −1 PRF reporter containing an impaired upstream attenuation hairpin (6BPGC5′ WT) and a downstream SARS-CoV pseudoknot stimulator in the presence of different amounts of antisense (Restore DNA) in Supplementary Figure S1B. The 0 and −1 frame products are labeled as indicated. (**D**) Relative frameshifting activity of (C) with the frameshifting efficiency of reporter without restore DNA addition being treated as 1. The statistical analysis and designation are the same as those in (B).

We have demonstrated that the formation of an upstream attenuator hairpin can be controlled by alternate base-pairing schemes to achieve −1 PRF activity regulation (31). Aiming for sequence-specific regulation of −1 PRF, we designed DNA oligonucleotides complementary to the 5′-half (6BPGC-5′-DNA) or the 3′-half (6BPGC-3′-DNA) sequences of the stem of a potent −1 PRF attenuator hairpin (6BPGC) (29) (Supplementary Figure S1A) to interfere with attenuation hairpin refolding. Each antisense DNA was designed to form either 19 or 20 bp with its complementary upstream RNA target to generate RNA–DNA duplexes of similar stability (37). We then measured the effect of each antisense on attenuation efficiency of a −1 PRF reporter (6BPGC-SARSPK) containing the upstream 6BPGC attenuator hairpin and a downstream SARS-CoV −1 PRF pseudoknot stimulator (30). The −1 PRF attenuation was tracked by decreased −1 PRF efficiency, which was observed from decreased −1 frame or/and increased 0 frame translation products upon antisense addition in a −1 PRF assay. Interestingly, addition of 6BPGC-5′-DNA resulted in a dose-dependent loss of 6BPGC attenuator activity consistent with antisense-mediated attenuation hairpin disruption, whereas addition of 6BPGC-3′-DNA did not suppress attenuator activity of 6BPGC (Figure 1A and B). This means that −1 PRF activity can be oppositely controlled in-trans by antisense DNA oligonucleotides designed to target either side of the stem region of an upstream attenuator hairpin, providing a way to sequence-specifically regulate −1 PRF.

### Attenuation of −1 PRF by trans-formed upstream duplexes proximal to the slippery site

One possible explanation of the observed opposite effects in attenuation activity modulation between the two antisense oligonucleotides is that the accessibility for trans-duplex formation is different between the two sides of the refolding hairpin stem in the presence of a nearby ribosome. Alternatively, the opposite effects may have been caused by the difference in spacing from the slippery site between the two antisense-mediated RNA–DNA duplexes, given that proximity plays an important role in the attenuation efficiency of a cis-formed attenuator hairpin (29). Consistent with the later explanation, addition of an antisense DNA (restore DNA), with sequences complementary to the 3′-stem of an impaired attenuator hairpin (6BPGC5′WT) (Supplementary Figure S1B), led to enhanced attenuation of −1 PRF efficiency of a reporter (6BPGC5′WT-SARSPK1) containing 6BPGC5′- WT in a dose-dependent manner (Figure 1C and D). As the impaired attenuator hairpin shares the same 3′-stem sequences to those of 6BPGC, this result also rules out the accessibility issue. Thus, these findings indicate that an upstream duplex needs to be proximal to the slippery site for efficient −1 PRF attenuation and suggest that the duplex in a proximal upstream hairpin stem is the functional unit responsible for −1 frameshifting attenuation.

### Efficient attenuation requires longer upstream duplexes formed in-trans with ribosomal drop-off playing a minimal role in the observed −1 PRF attenuation

**Figure 2.**
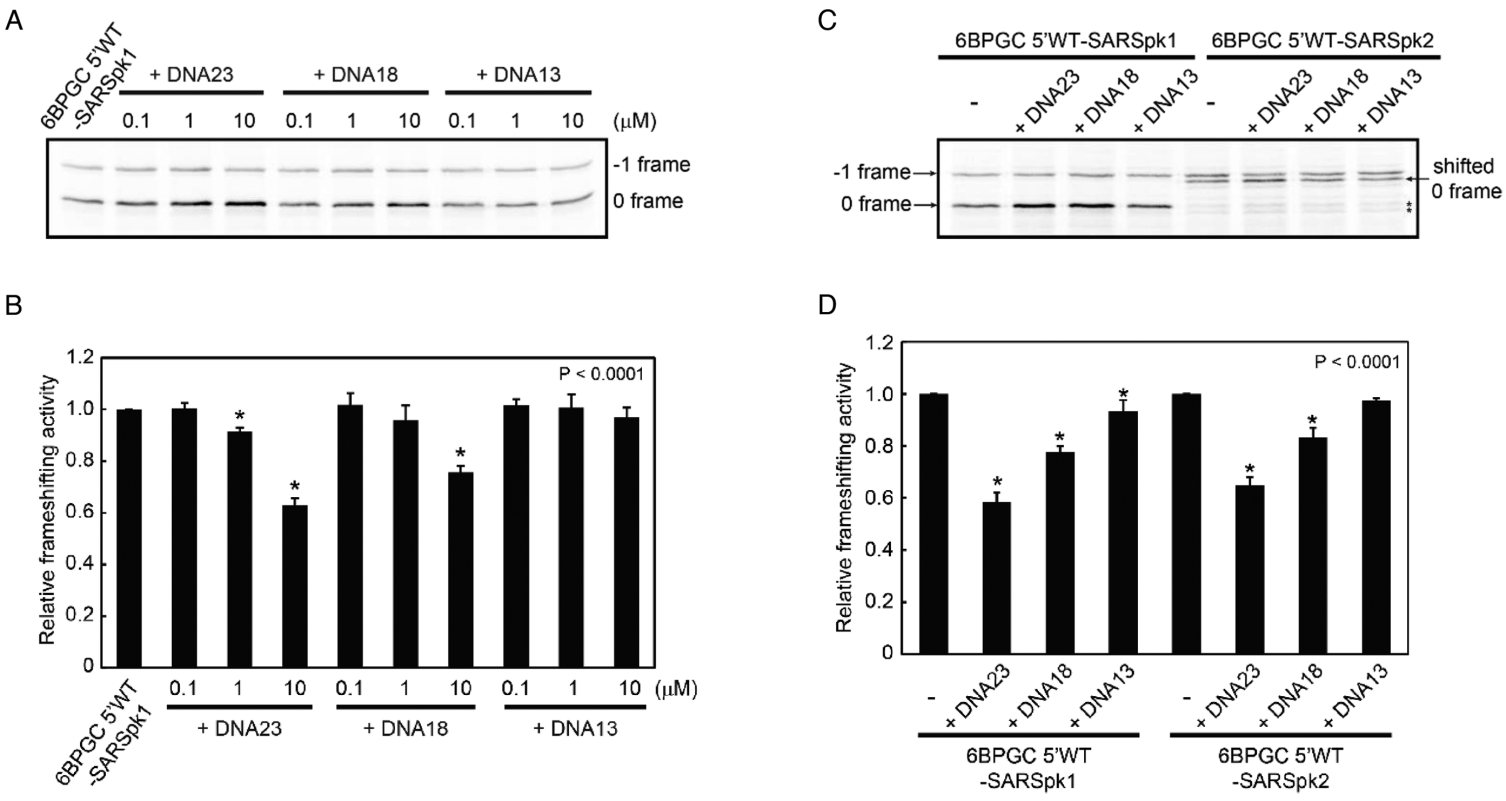
−1 PRF can be attenuated by antisense DNA-mediated upstream duplex of sufficient lengths formed in-trans. (**A**) SDS-PAGE analysis of ^35^S methionine-labeled translation products in reticulocyte lysate using the same reporter as that in Figure 1C in the presence of three different antisense DNA oligonucleotides (see Supplementary Figure S1C). The 0 and −1 frame products are labeled as indicated. (**B**) Relative frameshifting activity of (A) with the frameshifting efficiency of reporter without antisense DNA addition being treated as 1 for comparison. Value for each bar is the mean of three independent experiments with standard error of the mean. *P-*values were determined by a student's *t-*test with *P-*value < 0.0001 designated by an ‘*’ and referring to the comparison with the construct without the addition of an antisense. (**C**) SDS-PAGE analysis of ^35^S methionine-labeled translation products in reticulocyte lysate for two −1 PRF reporter constructs of different 0-frame stop codon positions in the presence of 10 μM of antisense DNA oligonucleotides (in Supplementary Figure S1C and D). The 0 and −1 frame products are labeled as indicated. Star signs indicate the potential drop-off products although both products appear in the absence of antisense. (**D**) Relative frameshifting activity of (C) with the frameshifting efficiency of reporter without antisense DNA addition being treated as 1. The statistical analysis and designation are the same as those in (B).

To see if there is a minimal requirement for duplex length of an effective upstream attenuation duplex, three antisense variants (restore DNA23, restore DNA18 and restore DNA13) with the potential of forming RNA–DNA duplex of 23, 18 and 13 bp were designed with spacing to the 0-frame E site being kept as 0 to prevent E-site invasion occurring (38) (Supplementary Figure S1C). The −1 PRF attenuation activity of the shortest upstream duplex declined dramatically while being compared with that of the longest upstream duplex (both mediated by 10 μM of antisenses) (Figure 2A and B). Although the proximity requirement of a functional upstream −1 PRF attenuation duplex in Figure 1A and B suggests that the observed −1 PRF attenuation is not the result of ribosomal drop-off during translational elongation of a ribosome, the loss of observed −1 PRF attenuation activity by a shorter upstream duplex in Figure 2A and B implies that the observed ‘−1 PRF attenuation’ by restore DNA23 could have been caused by drop-off effect mediated by the longer upstream duplex (39). In particular, the gel based assay may not resolve the potential upstream duplex-mediated drop-off product from the 0-frame translation product. Eventually, this could result in the amount of 0-frame product being overestimated, leading to underestimation of −1 PRF efficiency. To address this issue, we created a new construct (6BPGC5′WT-SARSPK2) with the 0-frame stop codon being moved further downstream of the slippery site (Supplementary Figure S1D) to help distinguishing 0-frame products from the potential drop-off products that should appear within upstream mRNA sequences targeted by the antisenses. The trend in −1 PRF attenuation efficiency for upstream duplexes of different lengths using the 6BPGC5′WT-SARSPK2 construct is similar to that of the one used in Figure 2B in the presence of 10 μM of antisenses (Figure 2C and D), indicating upstream duplexes of sufficient length do act as a −1 PRF attenuator. We noted that the attenuation efficiencies of antisense-mediated upstream duplexes in Figures 1 and 2 were not dramatic (but statistically significant) in the presence of 1 and 10 μM of antisenses because they were calculated from constructs carrying 6BPGC5′WT hairpin that possesses residual −1 PRF attenuation activity. Nevertheless, these results confirm that the −1 PRF attenuation effect can be generated in-trans by an antisense DNA-mediated upstream duplex proximal to the slippery site.

### Upstream RNA–DNA duplexes attenuate −1 PRF stimulated by distinct downstream stimulators

**Figure 3.**
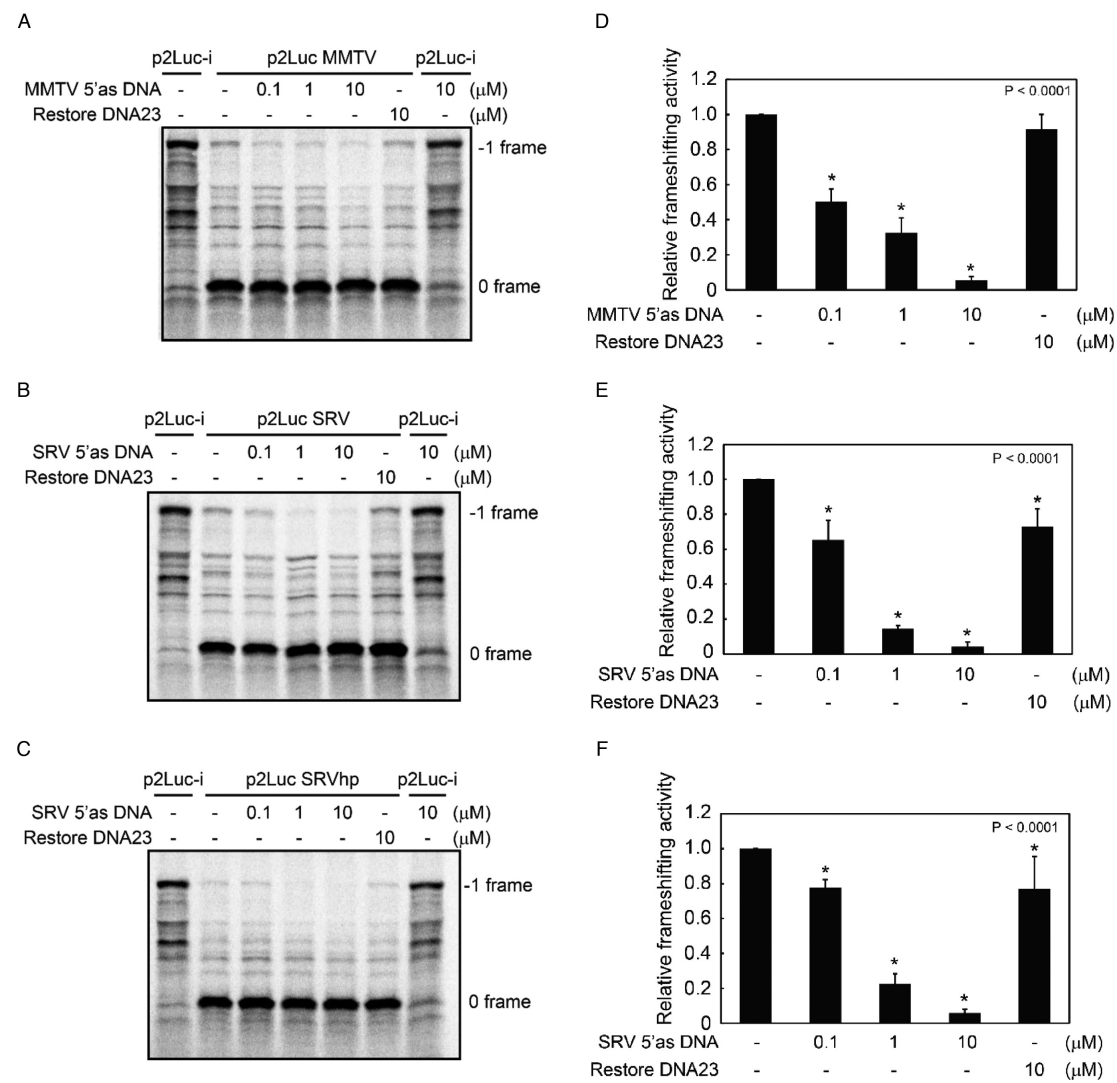
Antisense DNA-mediated upstream duplexes attenuate the −1 PRF activity stimulated by several distinct −1 PRF stimulators *in vitro*. (**A**–**C**) *In vitro* radioactivity based −1 PRF assays performed in reticulocyte lysate using full-length p2luc reporters containing distinctive types of stimulator (as shown in Supplementary Figure S2) in the presence of different amounts of corresponding antisense DNA targeting upstream sequences. The stimulator used in (A) is MMTV pseudoknot while the stimulators used in (B) and (C) are SRV pseudoknot and SRV hairpin, respectively. The p2luci vector was used as the −1 frame product control in all three cases. (**D**–**F**) Relative frameshifting activity of (A–C). Relative frameshifting activity was calculated with the frameshifting efficiency of reporters without antisense DNA addition being treated as 1 for comparison. Value for each bar is the mean of three independent experiments with standard error of the mean. *P-*values were determined by a student's *t-*test with *P-*value < 0.0001 designated by an ‘*’ and referring to the comparison with the construct without the addition of an antisense or restore DNA23.

Previously, we have shown that an upstream −1 PRF attenuation hairpin could downregulate −1 PRF stimulated by distinct downstream stimulators (29). To see if the upstream duplex can be used to attenuate viral −1 PRF stimulators other than that of SARS-CoV, we compared the frameshifting efficiencies of several viral −1 PRF pseudoknot stimulators, including mouse mammary tumor virus (MMTV), simian retrovirus (SRV) and a hairpin stimulator derived from SRV pseudoknot (40–42) (Supplementary Figure S2), in the presence of different amount of antisense targeting upstream sequences (Supplementary Figures S2 and S3). Surprisingly, we found that significant −1 PRF attenuation was observed in the presence of 1 μM of antisenses by comparing with those in Figures 1 and 2. It could be explained by the less stable structures formed upstream of the slippery sites in the current reporters. By contrast, the amount of −1 frame product translated from a read-through control (p2luci) was not affected. Furthermore, the −1 PRF activities can be attenuated to different extents by designed antisense DNA of different complementarities to the sequences upstream of the slippery site. For example, 10 μM of restore DNA23 was needed to attenuate −1PRF to similar extent as that of 0.1 μM of SRV 5′ as (Figure 3E and F) because restore DNA23 forms less base pairs with the upstream sequences. Taken together, these results indicate that the upstream duplex approach provides a general means of downregulating −1 PRF activity stimulated by distinct types of downstream stimulators *in vitro*.

### Trans-added antisense DNA converts a weak viral attenuation element into a stronger attenuator

**Figure 4.**
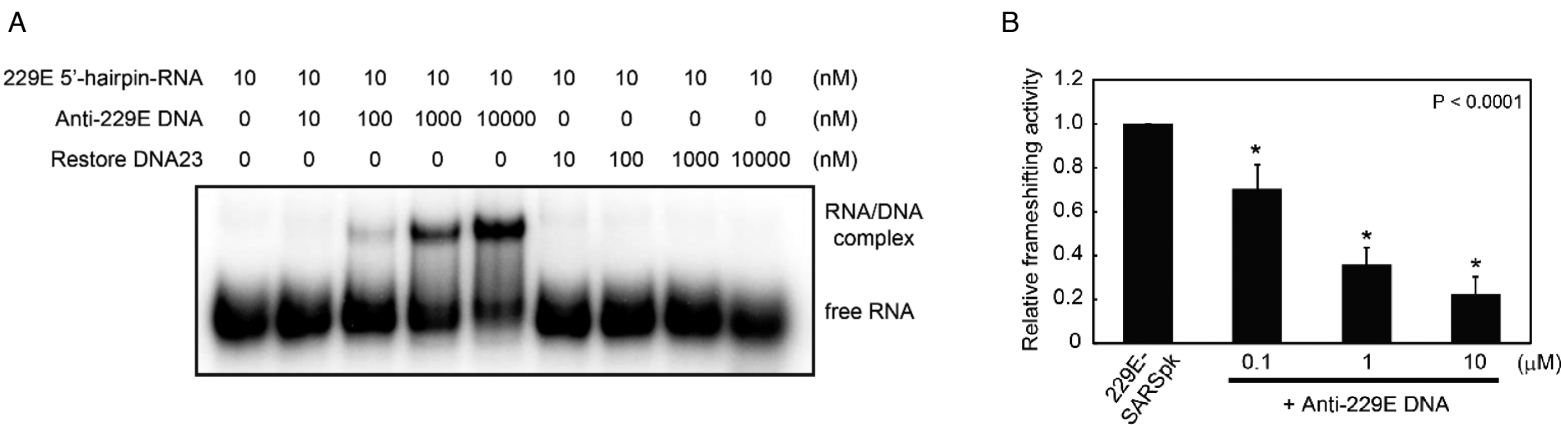
Pre-existed upstream conformation of weak attenuation activity can be converted into a stronger attenuator by the addition of an antisense DNA. (**A**) EMSA result of ^32^P-labeled 229E upstream hairpin RNA probe (see the sequences in Supplementary Figure S4D) in the presence of different amounts of unlabeled antisense DNA (anti-229E) (Supplementary Figure S4E) or restore DNA23 as the control. The free RNA and complex formed are labeled as indicated. (**B**) Relative frameshifting activity of the −1 PRF reporter in Supplementary Figure S4E in the presence of different amounts of anti-229E DNA, calculated from dual-luciferase assays in reticulocyte lysate with the frameshifting efficiency of reporter without antisense DNA addition being treated as 1. Value for each bar is the mean of three independent experiments with standard error of the mean. *P-*values were determined by a student's *t-*test with *P-*value < 0.0001 designated by an ‘*’ and referring to the comparison with the construct without the addition of anti-229E DNA.

To explore the potential of upstream duplex-mediated −1 PRF attenuation in anti-viral applications in human coronavirus (hCoV), we analyzed sequences upstream of the slippery sites of six known hCoVs and found a potential hairpin stem upstream of the −1 PRF slippery site of 229E-CoV in addition to the one characterized in SARS-CoV (Supplementary Figure S3A and B). However, the −1 PRF activity of a shortened p2luc reporter containing the 229E upstream hairpin and an SARS-CoV pseudoknot was not attenuated as efficiently as that by the SARS-CoV attenuator hairpin (Supplementary Figure S4A–C), and could be explained by the less stable free energy of the 229E upstream hairpin predicted by Mfold (43). We then asked if an antisense-mediated duplex can compete with the predicted 229E upstream hairpin to generate a potent −1 PRF attenuator in a duplex form. EMSA experiments indicated an isolated 229E upstream RNA hairpin (Supplementary Figure S4D) forming a complex with an antisense DNA (anti-229E) that targets the 3′-side sequences of the hairpin (Supplementary Figure S4E), suggesting the formation of an RNA–DNA duplex (Figure 4A). Furthermore, the addition of anti-229E led to higher −1 PRF attenuation activity than the cis-formed 229E viral attenuator hairpin alone in a full-length p2luc-based −1 PRF reporter (Supplementary Figure S4E and S4B). Thus, pre-existing local conformations upstream of −1 PRF slippery site can be programmed by the antisense approach to generate a functional −1 PRF attenuator in the form of a trans-formed duplex.

### An upstream attenuation duplex efficiently downregulates −1 PRF activity stimulated by multiple −1 PRF signals of MERS-CoV in human 293T cell lysates

**Figure 5.**
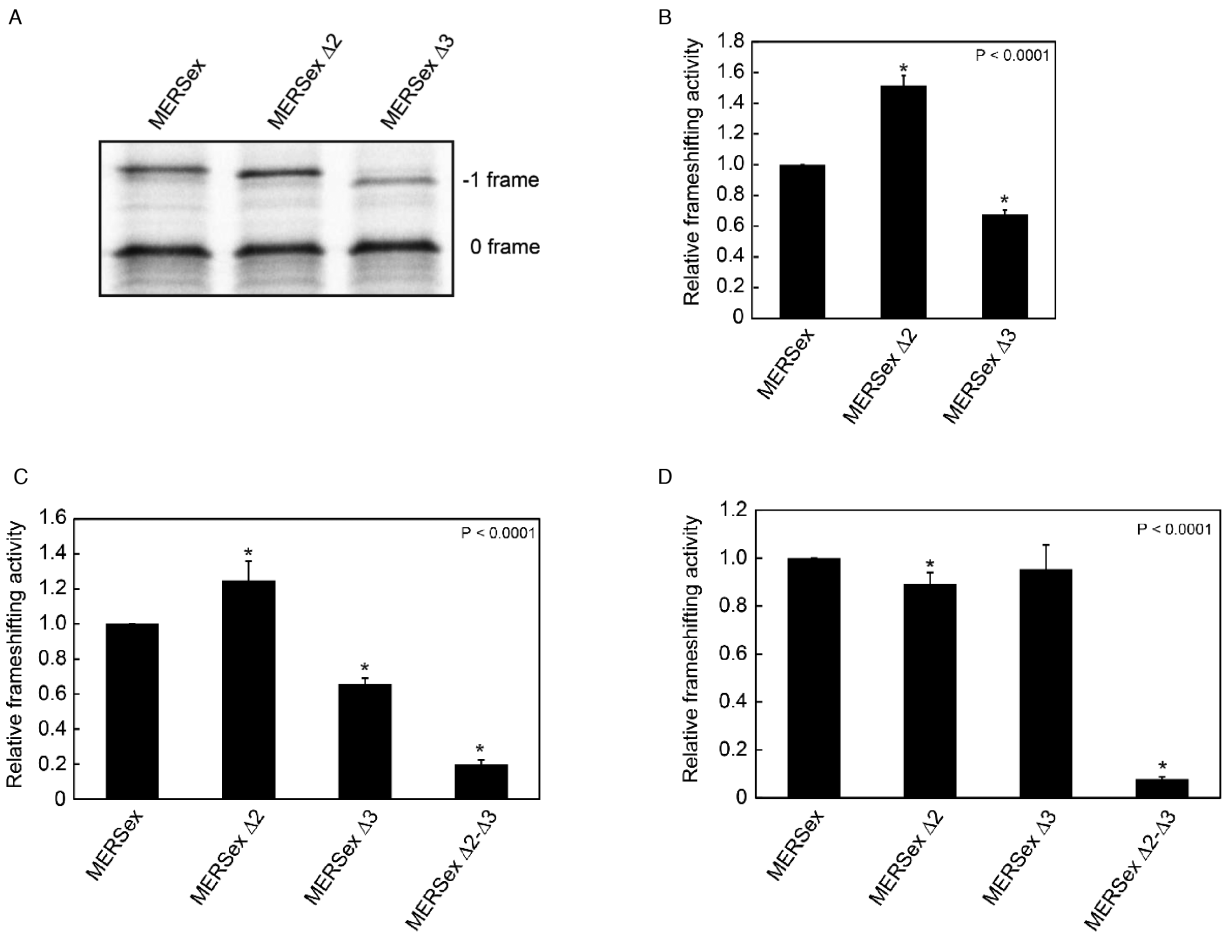
Two alternative pseudoknot stimulators formed by viral sequences downstream of the UUUAAAC slippery sequences of MERS-CoV possess substantial −1 PRF activity. (**A**) SDS-PAGE analysis of ^35^S methionine-labeled translation products in reticulocyte lysate for the three different MERS-CoV viral variant sequences in Supplementary Figure SS6A–C using the shortened p2luc −1 PRF reporter. (**B**) The relative frameshifting efficiency of (A) with the frameshifting efficiency of the reporter containing MERSex sequence being treated as 1. Value for each construct is the mean of three independent experiments with standard error of the mean. *P-*values were determined by a student's *t-*test with *P-*value < 0.0001 designated by an ‘*’ and referring to the comparison with the MERSex construct. (**C**) Relative frameshifting efficiency calculated from dual-luciferase activity measured in reticulocyte lysate for different MERS-CoV viral deletion sequences (Supplementary Figure S6A–E) inserted in the full-length p2luc −1 PRF reporters, with the frameshifting efficiency of the reporter containing MERSex sequence being treated as 1. Value for each construct is the mean of three independent experiments with standard error of the mean. *P-*values were determined by a student's *t-*test with *P-*value < 0.0001 designated by an ‘*’ and referring to the comparison with the MERSex construct. (**D**) Relative frameshifting efficiency of different MERS-CoV viral deletion sequences in (C) calculated from dual-luciferase activity measurement of transfected human 293T cells, with the frameshifting efficiency of the reporter containing MERSex sequence being treated as 1. The statistical analysis and designation are the same as those in (C).

**Figure 6.**
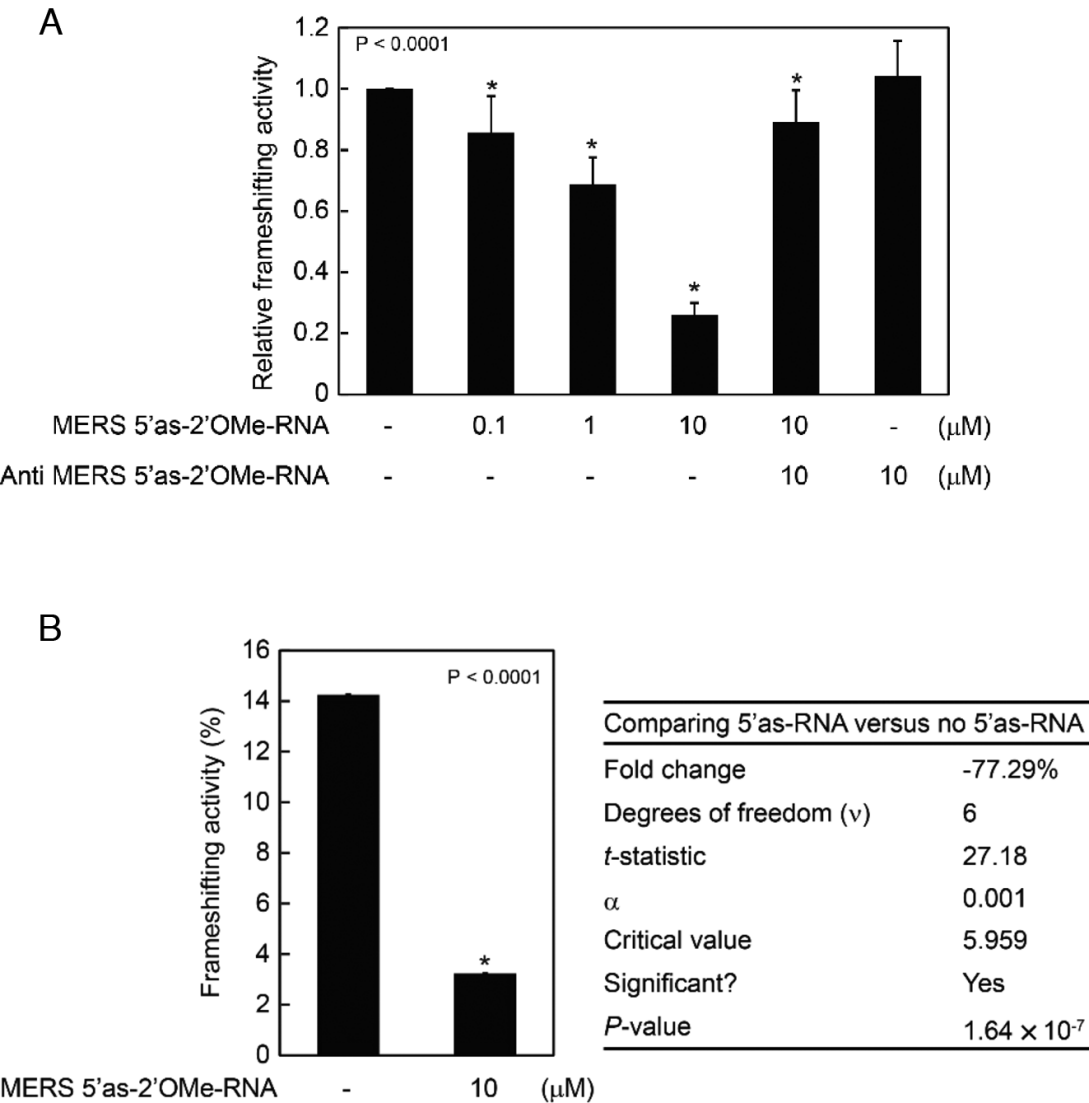
An upstream duplex mediated by 2′ OMe-modified RNA antisense oligonucleotide attenuates −1 PRF activity from multiple −1 PRF signals of MERS-CoV in human cell lysate *in vitro*. (**A**) Relative frameshifting activity of a p2Luc reporter harboring MERS-CoV viral −1 PRF sequences, MERSex (nucleotides 13 382–13 627) (48) in the presence of different amounts of 2′ OMe-modified RNA antisense and anti-antisense control (Supplementary Figure S6). The frameshifting assays were conducted in human 293T cell lysate using capped mRNA obtained by *in-vitro* transcription. The dual-luciferase activity was measured to calculate the *in vitro* frameshifting efficiency with the frameshifting efficiency of the reporter without antisense addition being treated as 1. Value for each bar is the mean of three independent experiments with standard error of the mean. *P-*values were determined by a student's *t-*test with *P-*value < 0.0001 designated by an ‘*’ and referring to the comparison with the construct without the addition of a 2′ OMe-modified RNA. (**B**) Summary of unpaired two-sample t-test for the effects of MERS 5′ as-2′ OMe-RNA on MERSex −1 PRF frameshifting activity. Calculated frameshifting activity for MERSex against a read-through control (Supplementary Figure S6) with or without 10 μM of 2′ OMe-modified RNA antisense in the same condition as those in (A). The data was statistically analyzed by a procedure suggested for bicistronic reporter assay (36) with results summarized in the right panel.

An antisense approach has been applied to disrupt the downstream −1 PRF stimulator of SARS-CoV (26). However, the success of this approach requires characterization of the boundary of the downstream stimulator. Such information may not be available for a new outbreak pathogen, such as the MERS-CoV (27). Indeed, we found that both a SARS-CoV type 3-stems pseudoknot (28,44–46) and a 229E type kissing hairpin pseudoknot (47) might exist downstream of the UUUAAAC −1 PRF slippery site of the MERS-CoV (48) (Supplementary Figure S5A and B). Additionally, two −1 PRF reporters containing sequence deletions of MERS-CoV −1 PRF signal that block the formation of either pseudoknot (Supplementary Figure S6) possessed substantial −1 PRF activity both *in vitro* and in 293T cell (Figure 5 A–D), suggesting that both pseudoknots may function during viral replication although the contribution of either pseudoknot to viral −1 PRF activity requires further study. 2′ OMe-modified RNA was used to evaluate the viral −1 PRF attenuation activity of the antisense-mediated upstream duplex in human cell lysate because DNA antisense would be digested in crude cellular lysates. We found that a 2′ OMe-modified antisense RNA oligonucleotide capable of mediating upstream duplex formation (Supplementary Figure S6A) efficiently downregulated the *in vitro* −1 PRF activities of the MERS-CoV viral sequence capable of forming alternative downstream stimulators in a dosage-dependent manner in human 293T cell lysate, whereas the attenuation effect was neutralized by the co-existence of an anti-antisense (Supplementary Figure S6A and Figure 6A). A more rigorous statistical analysis recommended for p2-luc based assay (36) using 10 μM of 2′ OMe-modified antisense RNA and a read-through control led to a similar extent of inhibition (Figure 6B). Therefore, targeting the sequence upstream of the slippery site represents an efficient and straightforward approach to viral −1 PRF inhibition because detailed knowledge of the downstream stimulator boundary is not required. This approach should be useful in looking for quick therapeutic solutions to emerging pathogens such as the MERS-CoV.

## DISCUSSION

### Potential mechanisms of −1 frameshifting attenuation

A stable structure could act as a roadblock to cause ribosomal drop-off in addition to stimulating −1 PRF. Accordingly, a frameshifting pseudoknot can prohibit a significant fraction of frameshifted ribosomes that it stimulated from reaching the −1 frame stop codon. Eventually, it leads to the compromise of observed −1 PRF efficiency (49). By contrast, the trans-formed −1 PRF attenuator duplex identified in this work is upstream of the slippery site and the potential ribosomal drop-off effect mediated by the duplex should occur before the ribosome reaches the slippery site. While the drop-off effect could lead to under-estimation of −1 PRF efficiency if the drop-off products could not be distinguished from the 0-frame products, the observations from experimental design that moves the 0-frame stop codon away from the location of upstream duplex (in Figure 2C and D) clearly clarified the role of upstream duplexes in −1 PRF attenuation. Furthermore, both a cis-formed upstream hairpin (29) and a trans-formed duplex (this work) can act to attenuate −1 PRF stimulated by a variety of downstream stimulators in 80S ribosome systems, suggesting both attenuators could share the same −1 PRF attenuation mechanism.

Previously, a ribosomal E site adjacent duplex formed between an internal SD-like element upstream of the slippery site and the anti-SD sequence of 16S rRNA was shown to attenuate prokaryotic −1 frameshifting efficiency in *E. coli* (18). By contrast, internal SD can promote release factor 2 (RF2)-dependent +1 frameshifting when placed adjacent to the ribosomal E site (20). Such an opposite role in −1 and +1 frameshifting regulation led to the suggestion of a tension-mediated mechanism in frameshifting stimulation (19). Alternatively, such effects could be caused by the elongation pausing mediated by an internal SD·anti-SD interaction during the elongation of 70S ribosome (50). Interestingly, we have previously shown that an upstream hairpin can stimulate +1 frameshifting in yeast in addition to attenuating −1 PRF in an *in vitro* 80S translation system (29). The mechanism of *in-cis* refolding hairpin as well as trans-formed upstream duplex in eukaryotic frameshifting regulation may thus be relevant to that of the duplex formed between internal SD and 16S rRNA in prokaryotic frameshifting regulation, given that formation of a duplex upstream of the slippery site is involved in all these cases. However, moving the attenuator hairpin 5′ further (29) did not enhance −1 PRF efficiency in the same manner as SD-like stimulator element did (18). A possible reason for this difference is that the internal SD-mediated duplex involves the 16S ribosomal RNA component of 70S ribosome and is part of the translational machinery, whereas the eukaryotic ribosome lacks an anti-SD sequence. Due to such a difference between 70S and 80S translation systems, the antisense-mediated upstream duplex identified in this work could simply act as a wheel chock to block −1 ribosome movement triggered by downstream −1 PRF stimulator mediated tension effect (14–16).

The stimulation mechanism of −1 PRF was best analyzed in the 70S ribosome system (13,51–53) and single-molecule experiments have linked the existences of downstream −1 PRF stimulator structures to the modulation of several molecular events within a translocation cycle (51–53). Ongoing works will test if a trans-formed upstream duplex can replace the functionality of an internal SD·anti-SD interaction in the 70S ribosome for frameshifting regulation aimed for establishing a link between 70S and 80S ribosome in frameshifting regulation via upstream duplexes of different forms. The effects triggered by downstream stimulators during translocation cycle (51–53) could then be examined in the presence of an upstream duplex to help illustrating the mechanisms of both stimulation and attenuation.

### Antisense-mediated upstream −1 PRF attenuation duplex as an alternative antiviral strategy toward emerging human CoVs

Outbreak of the SARS-CoV at Asia in 2013 was followed by the emergence of MERS-CoV in the Kingdom of Saudi Arabia 10 years later. Both virologists and epidemiologists predict that there will be more and more novel human coronaviruses emerging due to rapid mutation of viral genomes and the zoonotic features (54–55). Unfortunately, there is no approved vaccine for SARS or MERS. Therefore, the development of an antiviral strategy for rapid response to the emerging coronavirus infection is important.

Antiviral approaches against viral −1 PRF have been targeting on the downstream stimulators (20–22). Although small-molecule drugs provide uptake advantage, detailed structural information of the stimulator RNA is needed for this approach. Unfortunately, no high resolution −1 PRF stimulator structure of hCoV is available. Recently, three-dimensional RNA structure modeling of SARS-CoV −1 PRF stimulator pseudoknot in combination with computer-aided drug library modeling and screening have helped identifying potential leads for the inhibition of SARS-CoV −1 PRF (25). However, the proposed binding pocket of the leads seems to be specific for SARS-CoV pseudoknot stimulator (25).

An alternative approach using complementary PNA to disrupt SARS-CoV −1 PRF stimulator pseudoknot has also achieved antiviral effects and suppressed the propagation of SARS-CoV replicon when tagged with a cell-permeable peptide that facilitates cellular delivery (26). However, such an approach still requires information on the viral −1 PRF stimulator boundary and may not be deliverable in time. By contrast, the upstream duplex approach provides a more straight-forward means of inhibiting −1 PRF dependent viral pathogens than targeting the viral downstream stimulator and should be applicable as soon as the slippery sequence information of an emerging hCoV is available. Furthermore, the finding of antisense DNA-mediated upstream duplex in −1 PRF inhibition in *in vitro* translation systems should make early stage analysis much more affordable and accessible because expensive modified oligonucleotides are not needed.

Although nucleic acid-based therapy is still in its infancy, recent positive results in applications of modified oligonucleotides to against expanded trinucleotide repeats in Huntington's disease *in vivo* (56) and to protect post-exposure of the lethal Ebola virus infection (57) have demonstrated its powerful potential as a therapeutic agent. The processes aimed at facilitating cellular delivery of nucleic acid-based therapeutic agents using different oligonucleotide modifications or lipid nanoparticles also show promising results (58–59). With doubt over the delivery of safe vaccines against coronaviruses (60–61), the ability to attenuate viral −1 PRF in-trans by antisense-mediated duplex upstream of a viral slippery site thus provides a general strategy to quickly inhibit viral −1 PRF and replication of emerging human coronaviruses.

## Supplementary Material

SUPPLEMENTARY DATA
