# Supplementary material for: A general strategy to inhibiting viral −1 frameshifting based on upstream attenuation duplex formation
Source: Nucleic Acids Res. 2015 Nov 26;44(1):256–66. doi: 10.1093/nar/gkv1307 (PMC4705660; doi:10.1093/nar/gkv1307)
Supplement: SUPPLEMENTARY DATA [file supp_44_1_256__index.html]

A general strategy to inhibiting viral −1 frameshifting based on upstream attenuation duplex formation — A general strategy to inhibiting viral −1 frameshifting based on upstream attenuation duplex formation — SUPPLEMENTARY DATA 

# A general strategy to inhibiting viral −1 frameshifting based on upstream attenuation duplex formation

## SUPPLEMENTARY DATA

- SUPPLEMENTARY DATA
