# Supplementary material for: A general strategy to inhibiting viral −1 frameshifting based on upstream attenuation duplex formation
Source: Nucleic Acids Res. 2015 Nov 26;44(1):256–66. doi: 10.1093/nar/gkv1307 (PMC4705660; doi:10.1093/nar/gkv1307)
Supplement: SUPPLEMENTARY DATA [file supp_gkv1307_nar-03037-f-2015-File003.pdf]

Supplementary Information

"A general strategy to inhibiting viral -1 frameshifting based on upstream attenuation duplex formation"

by Hao-Teng Hu[‡], Che-Pei Cho[‡], Ya-Hui Lin, and Kung-Yao Chang[★]

Institute of Biochemistry, National Chung-Hsing University, 250 Kuo-Kung Road, Taichung, 402 Taiwan

This supplement includes 3 supplementary figures, and is organized in the following order:

1. Supplementary Figures and Legends.
2. Supplementary references.

Supplementary Figures and Legends.

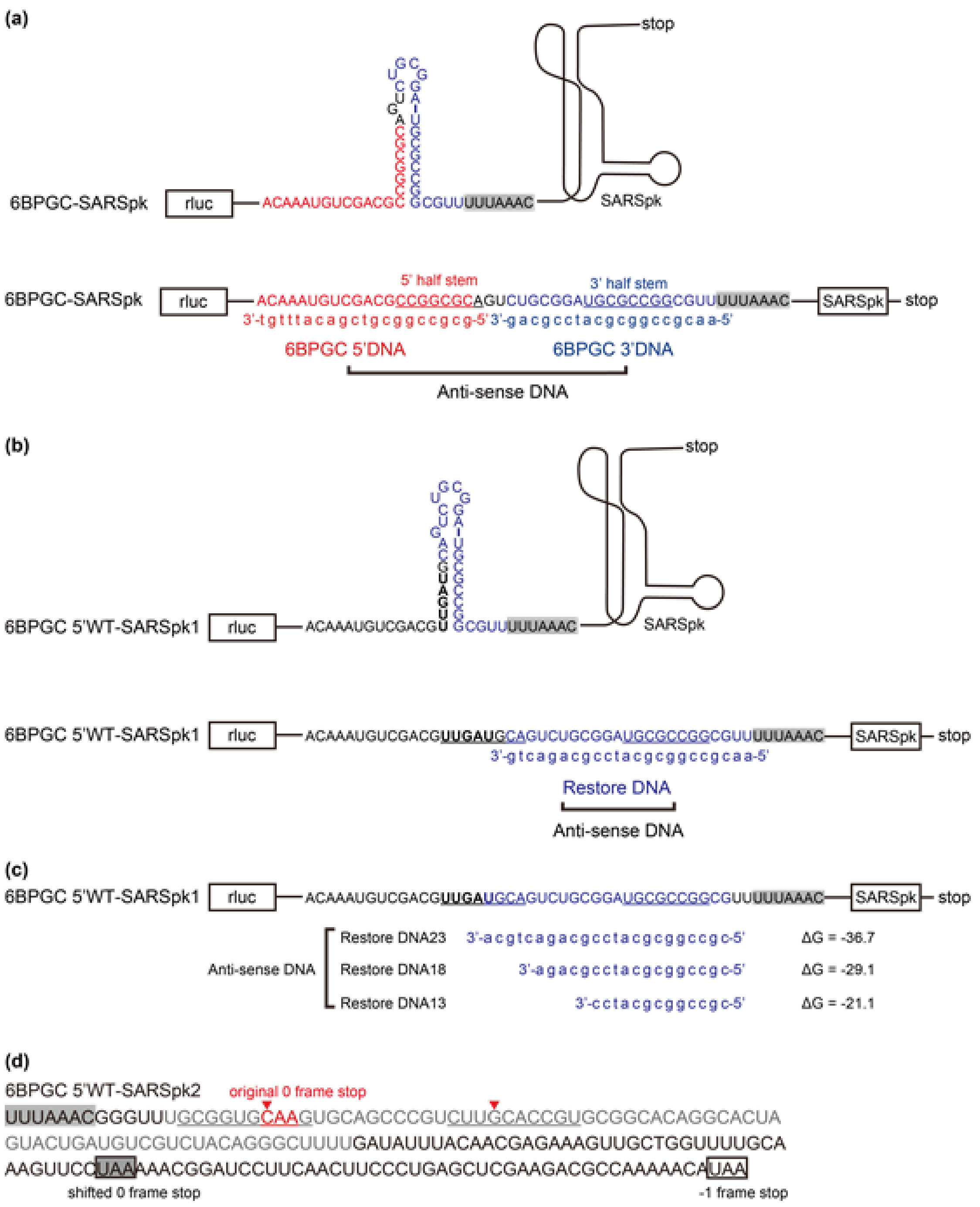

Supplementary Figure 1. The sequences of designed DNA antisenses and the sequences of hairpins upstream of UUUAAAC slippery site in shortened p2luc -1 PRF reporters used in Figures 1 and 2. (**A**) Sequences of upstream attenuation hairpin (6BPGC) in 6BPGC-SARSPK reporter construct and the designed antisense DNA oligonucleotides (typed in lower case) targeting the attenuation hairpin stem. The sequences involved in the formation of lower hairpin stem are underlined while the sequences targeted by the antisense oligonucleotides are colored with the same color as the corresponding antisense. (**B**) Sequences of an impaired upstream attenuation hairpin (6BPGC5'WT) in the 6BPGC5'WT-SARSPK1 reporter construct and the designed antisense (restore DNA) targeting the 3'-stem of 6BPGC5'WT. Please note that the 3'-stem sequences of 6BPGC5'WT are identical with those of 6BPGC attenuation hairpin in (A) with the sequences involved in the formation of an imperfect hairpin stem being underlined. (**C**) Sequences of the designed antisense DNA oligonucleotides (typed in lower case) targeting the upstream element of slippery site of 6BPGC5'WT-SARSPK1 construct with potential of forming duplexes of different lengths and the predicted free energy of the formed DNA-RNA duplex. Please note that the spacing toward the 0-frame E site for each duplex is kept as 0 to avoid E site invasion that may occurs for the upstream duplex mediated by restore DNA in (B). (**D**) The 0-frame and -1 frame junction sequences of a reporter construct (6BPGC5'WT-SARSPK2) derived from 6BPGC5'WT-SARSPK1 with the 0-frame stop codon moved downstream further from the slippery site. The original 0-frame stop codon UAA residing within Stem 1 (underlined) of the downstream SARS pseudoknot (sequences in gray color) was mutated to CAA (typed in red) with a complementary UUG sequence (both arrowed) to restore the base-pairing integrity of stem1. The new 0-frame stop codon is shaded and boxed, while the -1 frame stop codon (remained the same position as that in 6BPGC5'WT-SARSPK1) is indicated by an open box.

MMTV 5'as DNA
p2luc-MMTV-FL 3'-g a g t t t t t a c t t g t t t a c a g c t g t-5'
5'-AUCAAAUCGUUCGUUGAGCGAGUUCUCAAAAAUGAACAAAUGUCGACAUU
UUUAAACUUGUAAAGGGGCAGUCCCCUAGCCCCACUCAAAAGGGGGAAGGA
UCC-3'

SRV 5'as DNA
p2luc-SRV-FL 3'-t t t t t a c t t g t t t a c a g c t g c t g c-5'
5'-UUCGUUGAGCGAGUUCUCAAAAAUGAACAAAUGUCGACGACGUUUUUAAA
CUAGUUGAGCGGCCAGCUCCAGGCCGCCAAACAAUAUGGAGCAUGGAAGGA
UCC-3'

SRV 5'as DNA
p2luc-SRVhp-FL 3'-t t t t t a c t t g t t t a c a g c t g c t g c-5'
5'-UUCGUUGAGCGAGUUCUCAAAAAUGAACAAAUGUCGACGACGUUUUUAAA
CUAGUUGAGCGGCCUGGAGCUUCGGCUCCAGGCCGCCAAGGAUCC-3'

Supplementary Figure 2. Distinct -1 PRF stimulator sequences and the sequences upstream of UUUAAAC slippery site in full-length p2luc -1 PRF reporters used in Figure 3. Sequences of the viral -1 PRF stimulators of MMTV, SRV and an SRV-derived hairpin stimulator as well as the sequences upstream of the slippery site used in full-length p2luc -1 PRF reporters are shown. The 0-frame stop codon are typed in red while the sequences of the downstream stimulators are typed in gray. The antisense DNA oligonucleotides (in lower case) used and the upstream sequences targeted by antisenses are both typed in blue color.

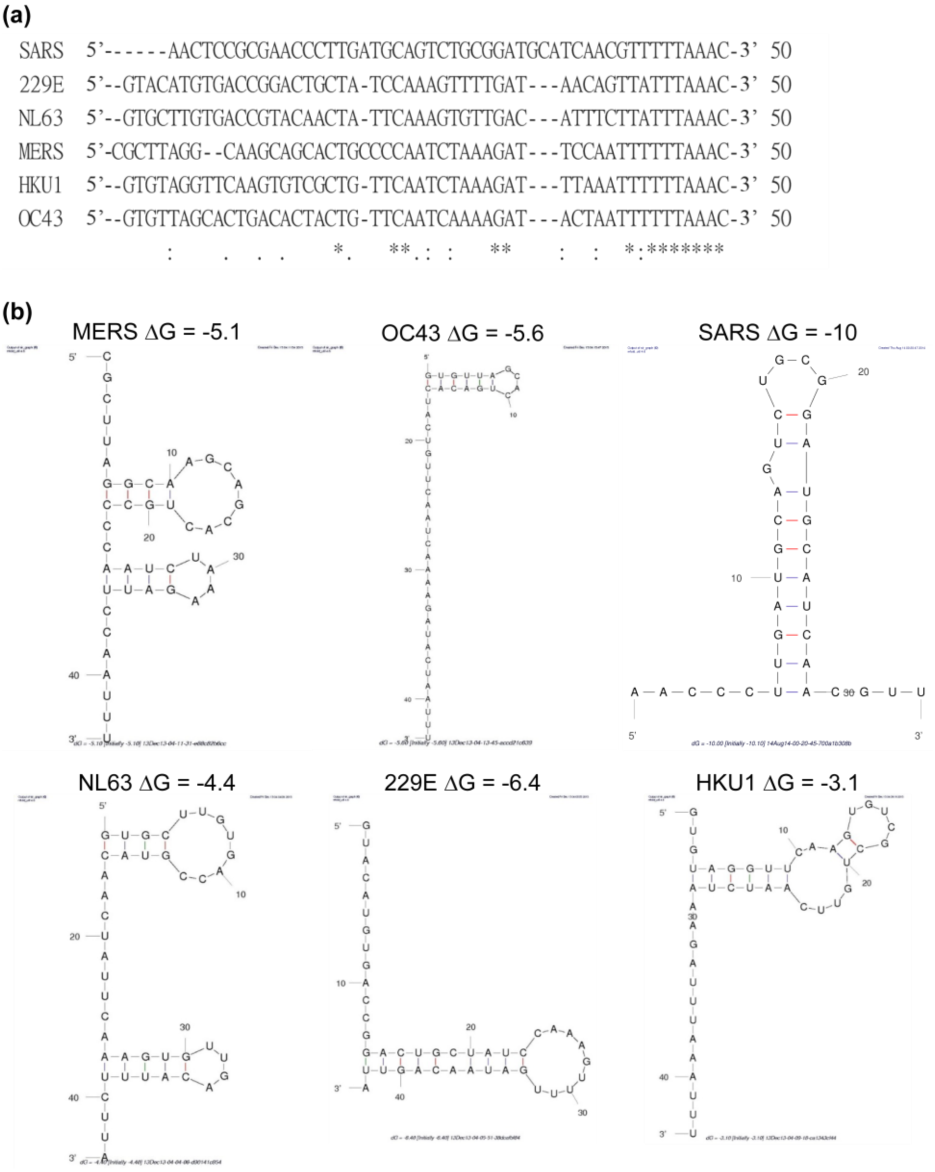

Supplementary Figure 3. The viral sequences as well as the predicted secondary structures upstream of -1 PRF slippery sites of six human coronaviruses. (**A**) Sequence alignment of the sequences upstream of UUUAAAC slippery sites of six human coronaviruses (1-6). (**B**) The predicted secondary structure and free energy of the six upstream viral sequences in (A) using Mfold (7).

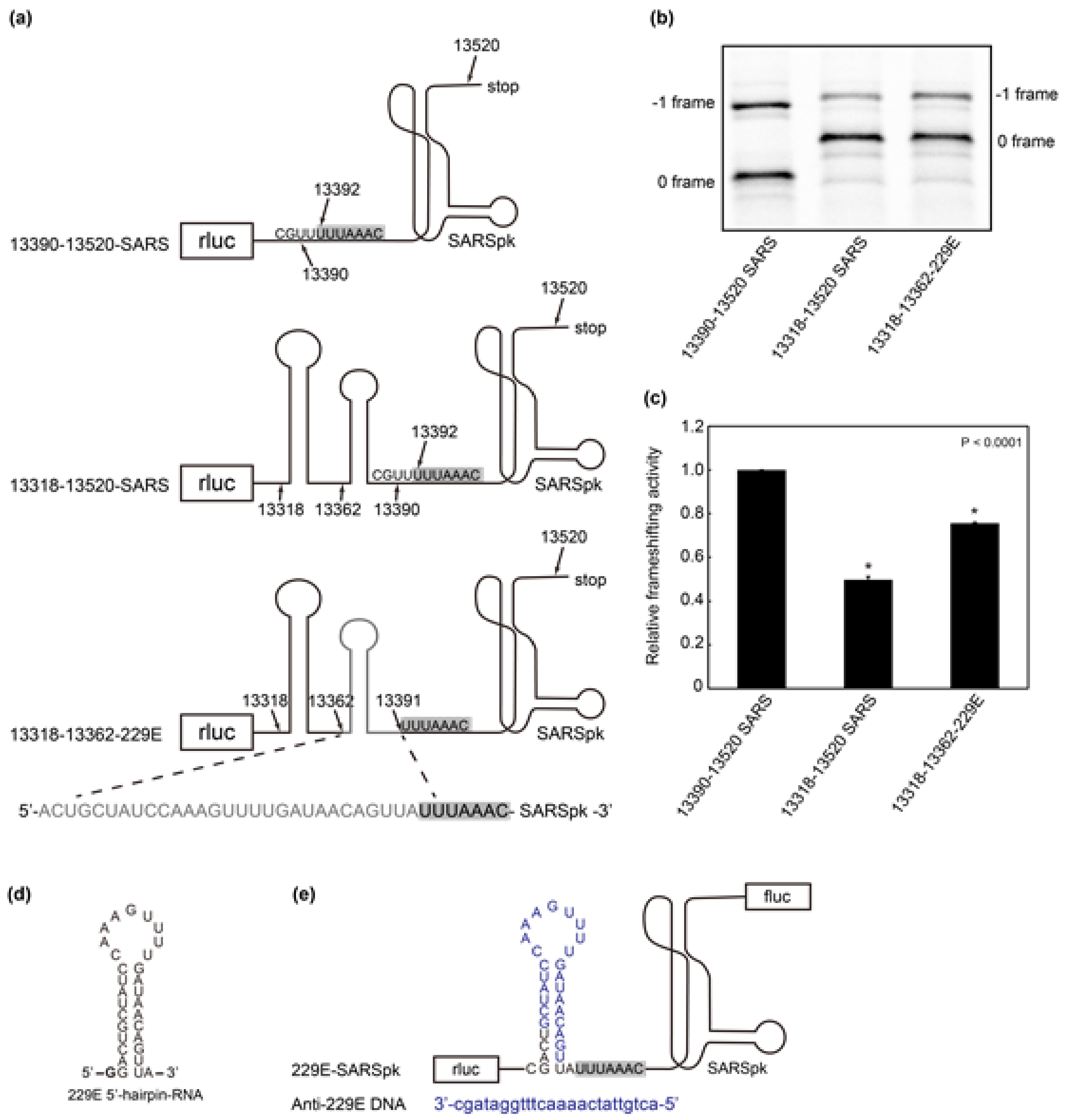

Supplementary Figure 4. The predicted upstream hairpin in 229E-CoV has weaker -1 PRF attenuation efficiency than that of the SARS-CoV. (**A**) Diagrams showing the replacement of SARS-CoV upstream attenuator hairpin with that of 229E CoV (the gray sequences in the bottom diagram) in a SARS-CoV -1 PRF stimulator-containing -1 PRF reporter using the SARS-CoV numbering system (8). (**B**) SDS-PAGE analysis of ^{35}S methionine-labeled translation products in reticulocyte lysate using a SARS-CoV pseudoknot-containing shortened p2luc -1 PRF reporter without (top diagram in (A)) or with upstream sequences from SARS-CoV (middle diagram in (A)) or with a chimeric sequence harboring the upstream hairpin of 229E-CoV (typed in gray) (bottom diagram in (A)). The 0 and -1 frame products are labeled as indicated. (**C**) Relative frameshifting activity of (B) with the frameshifting efficiency of reporter without upstream viral sequence, being treated as 1 for comparison. Value for each construct is the mean of three independent experiments with standard error of the

mean. *P*-values were determined by a student's *t*-test with *P*-value < 0.0001 designated by an * and referring to the comparison with the construct lacking an upstream hairpin. (**D**) The RNA sequences of 229E hairpin used for EMSA analysis in Figure 4A. The sequence has an extra G (typed boldly) in 5'-end to improve T7 RNA polymerase-based transcription yield. (**E**) A full-length p2luc-based -1 PRF reporter (229E-SARSPK) containing the 229E upstream attenuation hairpin and a downstream SARS-CoV pseudoknot stimulator was constructed to evaluate the effect of -1 PRF attenuation by antisense DNA. The antisense DNA (anti-229E) and its targeting sequences in 229E hairpin are both typed in blue color.

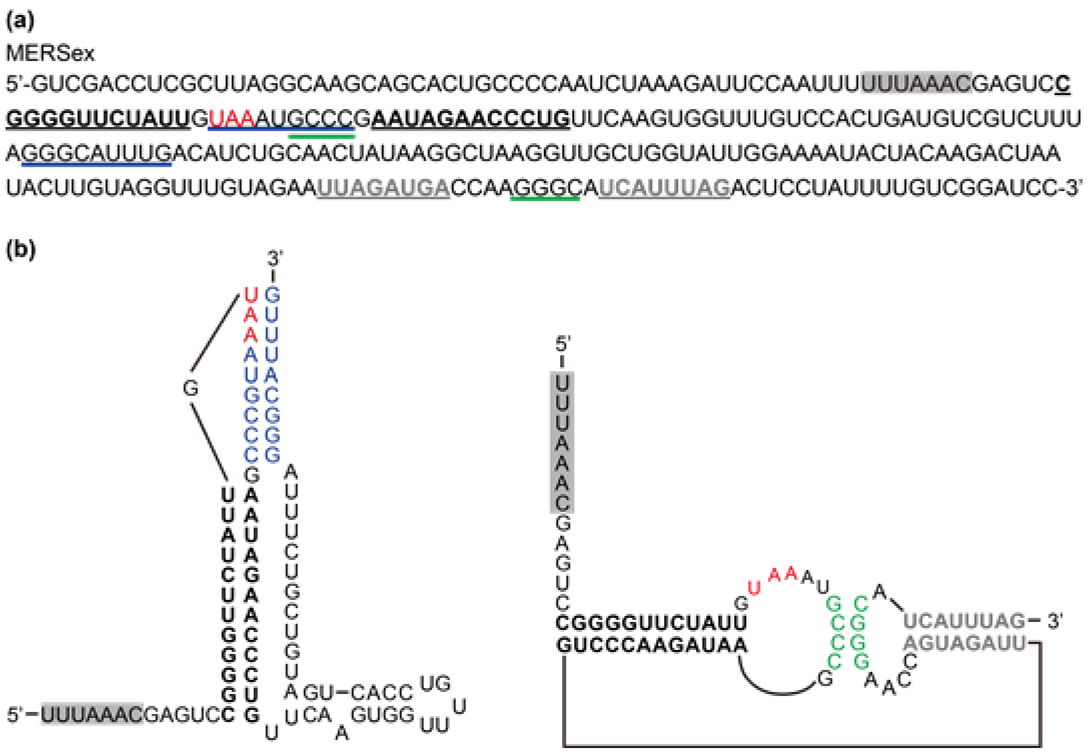

Supplementary Figure 5. Two types of pseudoknot stimulators could be formed by viral sequences downstream of the UUUAAAC slippery sequences of MERS-CoV. (**A**) Viral sequences flanking the UUUAAAC slippery site (shaded in gray) of MERS-CoV in MERSex construct with the potential base-pairing elements in the two types of pseudoknots underlined in blue (for 3-stem type) and green/gray (for kissing loop-type) colors, respectively. The stem 1 sequences shared by both pseudoknots are typed boldly with the 0-frame stop codon typed in red. MERSex was obtained by ligation-based PCR from synthetic DNA oligonucleotides according to the published MERS-CoV genomic sequences (6). (**B**) The schematic drawing for the two predicted pseudoknots downstream of slippery site in MERS-CoV according to the base-pairing schemes in (A).

(a)

5'-cacugccccaaucuaaagauucca-3'] Anti MERS 5'as-2'OMe-RNA

MERSex 3'-gugacgggguuagauuucuaaggu-5'] MERS 5'as-2'OMe-RNA

5'-GUCGACCUCGCUUAGGCAAGCAGCACUGCCCCAAUCUAAAGAUUCCAAUUUUUUAAACGAGUCC
GGGGUUCUAUUGUAAAUGCCCGAAUAGAACCCUGUUCAAGUGGUUUGUCCACUGAUGUCGUCUUU
AGGGCAUUUGACAUCUGCAACUAUAAGGCUAAGGUUGCUGGUAUUGGAAAAUACUACAAGACUAA
UACUUGUAGGUUUGUAGAAUUAGAUGACCAAGGGCAUCAUUUAGACUCCUAUUUUGUCGGAUCC-3'

(b)

MERSex Δ2

5'-GUCGACCUCGCUUAGGCAAGCAGCACUGCCCCAAUCUAAAGAUUCCAAUUUUUUAAACGAGUCC
GGGGUUCUAUUGUAAAUGCCCGAAUAGAACCCUGUUCAAGUGGUUUGUCCACUGAUGUCGUCUUU
AGGGCAUUUGACAUCUGCAACUAUAAGGCUAAGGUUGCUGGUAUUGGAAAAUACUACAAGACUAA
UACUUGUAGGUUUGUAGAAUUAGAUGACCAAGGGCAUCAUUUAGACUCCUAUUUUGUCGGAUCC-3'

(c)

MERSex Δ3

5'-GUCGACCUCGCUUAGGCAAGCAGCACUGCCCCAAUCUAAAGAUUCCAAUUUUUUAAACGAGUCC
GGGGUUCUAUUGUAAAUGCCCGAAUAGAACCCUGUUCAAGUGGUUUGUCCACUGAUGUCGUCUUU
AGGGCAUUUGACAUCUGCAACUAUAAGGCUAAGGUUGCUGGUAUUGGAAAAUACUACAAGACUAA
UACUUGUAGGUUUGUAGAAUUAGAUGACCAAGGGCAUCAUUUAGACUCCUAUUUUGUCGGAUCC-3'

(d)

MERSex Δ2-Δ3

5'-GUCGACCUCGCUUAGGCAAGCAGCACUGCCCCAAUCUAAAGAUUCCAAUUUUUUAAACGAGUCC
GGGGUUCUAUUGUAAAUGCCCGAAUAGAACCCUGUUCAAGUGGUUUGUCCACUGAUGUCGUCUUU
AGGGCAUUUGACAUCUGCAACUAUAAGGCUAAGGUUGCUGGUAUUGGAAAAUACUACAAGACUAA
UACUUGUAGGUUUGUAGAAUUAGAUGACCAAGGGCAUCAUUUAGACUCCUAUUUUGUCGGAUCC-3'

(e)

MERSex read-through control

5'-GUCGACCUCGCUUAGGCAAGCAGCACUGCCCCAAUCUAAAGAUUCCAAUUUGUUCAAGCGAGUCC
GGGGUUCUAUUGUAAAUGCCCGAAUAGAACCCUGUUCAAGUGGUUUGUCCACUGAUGUCGUCUUU
AGGGCAUUUGACAUCUGCAACUAUAAGGCUAAGGUUGCUGGUAUUGGAAAAUACUACAAGACUAA
UACUUGUAGGUUUGUAGAAUUAGAUGACCAAGGGCAUCAUUUAGACUCCUAUUUUGUCGGAUCC-3'

Supplementary Figure 6. Sequences of MERS-CoV -1 PRF variants used in Figures 5 and 6. (**A**) Viral sequences upstream of the UUUAAAC slippery sites of MERS-CoV in MERSex construct with the sequence targeted by an antisense colored in blue. A designed 2'OMe-modified RNA antisense (in blue) and an anti-antisense are both typed in lower case. (**B**) MERS-CoV -1 PRF viral sequence variant, MERSex Δ2, with the deletion of sequences that should block the formation of 3-stem type pseudoknot. (**C**) MERS-CoV -1 PRF viral sequence variant, MERSex Δ3, with the deletion of sequences that should block the formation of kissing-loop type pseudoknot. (**D**) MERS-CoV -1 PRF viral sequence variant, MERSex Δ2-Δ3, with the deletion of sequences that should block the formation of both 3-stem type and kissing-loop type pseudoknots (**E**) The sequences of read-through control used in Figure 6B. Three nucleotides (in red) were inserted to destroy the slippery sequence and shifting the reading frame into -1 frame.

Supplementary References

1. Thiel, V., Herold, J., Schelle, B. and Siddell, S.G. (2001) Infectious RNA transcribed in vitro from a cDNA copy of the human coronavirus genome cloned in vaccinia virus. *J. Gen. Virol.*, **82**. 1273-1281.

2. Vijgen, L., Keyaerts, E., Thoelen, I., Wollants, E., Lemey, P., Vandamme, A.M. and Van Ranst, M. (2005) Complete genomic sequence of human coronavirus OC43: Molecular clock analysis suggests a relatively recent zoonotic coronavirus transmission event. *J. Virol.*, **79**. 1595-1604.

3. He,R., Dobie,F., Ballantine,M., Leeson,A., Li,Y., Bastien,N., Cutts,T., Andonov,A., Cao,J., Booth,T.F., Plummer,F.A., Tyler,S., Baker,L. and Li,X. (2004) Analysis of multimerization of the SARS coronavirus nucleocapsid protein. *Biochem. Biophys. Res. Commun.*, **316**. 476-483.

4. van der Hoek,L., Pyrc,K., Jebbink,M.F., Vermeulen-Oost,W., Berkhout,R.J., Wolthers,K.C., Wertheim-van Dillen,P.M., Kaandorp,J., Spaargaren,J. and Berkhout,B. (2004) Identification of a new human coronavirus. *Nat. Med.*, **10**. 368-373.

5. Woo,P.C., Lau,S.K., Chu,C.M., Chan,K.H., Tsoi,H.W., Huang,Y., Wong,B.H., Poon,R.W., Cai,J.J., Luk,W.K., Poon,L.L., Wong,S.S., Guan,Y., Peiris,J.S. and Yuen,K.Y. (2005) Characterization and complete genome sequence of a novel coronavirus, coronavirus HKU1, from patients with pneumonia. *Biochem. J. Virol.*, **79**. 884-895.

6. van Boheemen, S., de Graaf, M., Lauber, C., Bestebroer, T.M., Raj, V.S., Zaki, A.M., Osterhaus, A.D., Haagmans, B.L., Gorbalenya, A.E., Snijder, E.J. and Fouchier, R.A.M. (2012) Genomic characterization of a newly discovered coronavirus associated with acute respiratory distress syndrome in humans. *mBio*, **3**. e00473-12

7. Zuker, M. (2003) Mfold web server for nucleic acid folding and hybridization prediction. *Nucleic Acids Res.*, **31**, 3406-3415.

8. Cho, C.P., Lin, S.C., Chou, M.Y., Hsu, H.T. and Chang, K.Y. (2013) Regulation of programmed ribosomal frameshifting by co-translational refolding RNA hairpins. *PLoS One*, **8**, e62283.
